# Unraveling overlapping deletions by agglomerative clustering

**DOI:** 10.1186/1471-2164-14-S1-S12

**Published:** 2013-01-21

**Authors:** Roland Wittler

**Affiliations:** 1 Genome Informatics, Faculty of Technology and Institute for Bioinformatics, Center for Biotechnology (CeBiTec), Bielefeld University, 33594 Bielefeld, Germany

## Abstract

**Background:**

Structural variations in human genomes, such as deletions, play an important role in cancer development. Next-Generation Sequencing technologies have been central in providing ways to detect such variations. Methods like paired-end mapping allow to simultaneously analyze data from several samples in order to, e.g., distinguish tumor from patient specific variations. However, it has been shown that, especially in this setting, there is a need to explicitly take overlapping deletions into consideration. Existing tools have only minor capabilities to call overlapping deletions, unable to unravel complex signals to obtain consistent predictions.

**Result:**

We present a first approach specifically designed to cluster short-read paired-end data into possibly overlapping deletion predictions. The method does not make any assumptions on the composition of the data, such as the number of samples, heterogeneity, polyploidy, etc. Taking paired ends mapped to a reference genome as input, it iteratively merges mappings to clusters based on a similarity score that takes both the putative location and size of a deletion into account.

**Conclusion:**

We demonstrate that agglomerative clustering is suitable to predict deletions. Analyzing real data from three samples of a cancer patient, we found putatively overlapping deletions and observed that, as a side-effect, erroneous mappings are mostly identified as singleton clusters. An evaluation on simulated data shows, compared to other methods which can output overlapping clusters, high accuracy in separating overlapping from single deletions.

## Introduction

It is well known that mutations in the human genome are associated to diseases such as cancer. Besides small scale mutations like single nucleotide variants, larger events such as deletions, insertions, inversions, or inter-chromosomal rearrangements can have a crucial impact on the initiation and development of cancer. The detection and analysis of these structural variations play an important role in understanding the underlying mechanisms of cancer, its diagnosis and treatment [1-4].

Current sequencing technologies allow to obtain high data volumes at low cost. It has now become affordable to sequence several samples of the same patient, enabling comparative analyses of, e.g., tumor cells versus healthy blood cells, or samples taken before versus after treatment, to distinguish tumor from patient specific variations, or to observe structural variations over time [5,6].

In the analysis of such complex data, it is important to consider heterogeneity of various kinds [7]. Besides the differences between several tissues or time points, in cancer one always has to face heterozygosity (mutations only affecting one allele), loss of heterozygosity (mutation inactivating the second allele), aneploidy (different copy numbers for some chromosomes), copy number alterations (different copy numbers for parts of chromosomes), differentiation of tumor cell lines developing different variations, etc. An additional challenge is that a cancer sample is most likely a mixed sample, i.e., although taken from tumor tissue, it usually contains also normal cells [2,8,9].

For the detection of single nucleotide variants (SNVs), there exist several approaches, some of which address the above issues. For instance, SomaticSniper [10], JointSNVMix [11] and MutationSeq [12] call somatic SNVs from pairs of normal and tumor samples, where the first two methods follow a Bayesian approach to distinguish tumor from patient specific SNVs, and the latter builds on clustering by support vector machines. Strelka [13] explicitly models mixtures of tumor and normal cells and can also call small indels. Also, several tools exist to accurately detect SNVs in pooled data [14-17], even mutations of low abundance. Apart from analyzing single SNVs, also haplotype inference and assembly has been addressed [18-20]. For the analysis of gene expression data, also Bayesian approaches have been proposed, even considering subtypes of cancer [21].

In contrast to the analysis of SNVs, for the detection of somatic deletions and other larger structural variations, one usually has to process the different samples separately and to compare the results afterwards, e.g., subtract deletions found in the healthy sample from those found in the tumor sample. Or one can pool the data and afterwards only select those calls solely based on tumor data [22]. Only recently, joint analysis of several data sets have been proposed [5,6].

As shown in a preliminary study [5] to detect deletions by a combined analysis of samples from tumor and healthy tissue, there were regions in the tumor genome for which existing tools predicted more deletions than there could actually be on a diploid genome. When instead two diploid sets of chromosomes were assumed, i.e., the tumor sample is actually a mixture of cancerous and healthy cells, almost all data could be explained consistently, by explicitly modeling heterozygous deletions on different alleles to be overlapping. These observations have been made particularly in regions where deletions were found in the healthy cells and additional deletions have been predicted in the tumor sample - thus in regions where it is especially difficult to distinguish cancer from patient specific mutations. However, the scope of the presented method has been to show that a consistent scenario of overlapping deletions can be found. It greedily constructs a "possible" solution, without raising the claim of reporting a "reasonable" result. Furthermore, the model is restricted to the very specific case of analyzing a mixture of cancer and normal cells, including some additional technical, combinatorial assumptions.

In this paper, we present a method to detect deletions that is particularly designed to handle overlapping deletions. For the sake of flexibility, no assumption on the composition of the data is made - whether it is just from one sample or pooled data, it contains different cell lines, aneploid cells, etc. Being aware of such heterozygosity and specially designed for such data, we refrain from predicting a deletion being "heterozygous or homozygous", or "tumor or patient specific". Instead, besides a tabular listing of the results, a rich visual output for each set of overlapping deletions is provided, allowing an easy inspection of the results.

The method takes as input paired ends that have been mapped to a reference genome and collects those mappings likely originating from the same deletion in clusters. Agglomerative clustering is utilized to cluster mappings by similarity. The similarity score is based on a three-dimensional representation of mappings and deletions similar to the two-dimensional representation introduced by Dew et al. [23] and used for structural variation detection by Sindi et al. [22] in the tool GASV. With this representation, we obtain a score that captures both the putative location and size of the deletion.

We applied our method to a data set from several samples taken from an acute lymphoblastic leukemia patient. Besides examples for predicted overlapping deletions, we find that single, putatively erroneous mappings not assigned to another cluster can nicely be identified as outliers. Since overlapping deletions are rare events and their verification is difficult, we performed an evaluation on simulated data showing good accuracy.

After providing the necessary background in the following section, we introduce our approach in Section "Method". In Section "Results and discussion", we present a simulation-based evaluation and results on real data, before we conclude our study.

## Background

We will now give a brief overview of existing approaches to identify structural variations and then introduce the technique of *paired-end mapping*, which our method is based on.

### Structural variation detection

Besides different experimental techniques, there are many computational approaches for structural variation detection [24]. A straight forward idea to detect mutations would be to fully assemble the genome under consideration, the so-called *donor genome*, and to align it to a reference sequence. To save the time and cost intensive assembly and finishing steps which would be necessary to determine the full genome sequence, one usually follows other approaches. Instead of performing a full assembly, one can restrict the process to only reads from regions suspect to harbor a variation, as for instance done in [25]. The tool *fermi *[26] allows both full assembly and a pre-filtering for reads unique to one sample. Other recent methods simultaneously assemble several genomes into a single graph data structure allowing for variation calling [27,28].

Another, more common approach is to omit any assembly of the donor genome and instead utilize the reads directly to detect differences by mapping them to the finished reference sequence. Basically, there are three classes of methods to identify structural variations from those mappings. (See [24] or [29] for reviews.) (1) Significant fluctuations of the coverage of the reference by mappings can indicate copy number changes. If part of a chromosome is lost or duplicated, the coverage drops or increases, respectively. (2) If a read has not been mapped completely, but in parts, this can indicate different types of mutations. E.g., if one half of the *split read *is mapped with some space to the remaining part, the segment in-between might be absent in the donor genome. If parts are mapped to different chromosomes, this can indicate inter-chromosomal rearrangements. (3) If the donor genome has been sequenced using a certain technology, pairs of reads (paired ends) are obtained. The orientation and the distance of the reads within a pair are known in the donor, and when the corresponding mappings on the reference do not agree with this pattern, structural variations can be called similar to the split read approach. This *paired-end mapping *technique, introduced by Korbel et al. [30], will be explained in detail in the following section.

Since all techniques have their advantages and disadvantages, in general, a combination of different techniques is advisable. Different tools can be applied separately and the results are combined in a post-processing step. Also tools exist which combine techniques already during the detection, e.g. inGAPsv [31], CNVer [32], GASVPro [33], SVseq2 [34], or the method by Nord et al. [35]. The method presented here is based on paired-end mapping. It is particularly designed for regions harboring several overlapping deletions. Due to the complexity of such regions, a partial assembly or coverage analysis is hardly possible. However, integrating a split read approach is planned for future work.

### Deletion detection by paired-end mapping

When genomic material from a sample is sequenced by short-read sequencing, many overlapping DNA-fragments are produced and a certain number of bases are read from both ends of each fragment, resulting in a pair of reads, so-called *paired ends*. The reading direction and the length of the reads are known. Further, since the fragment size is fixed - in practice distributed around the desired length - their approximate distance is known as well.

The paired ends from a newly sequenced donor genome can be mapped to a reference genome which is already assembled to a complete DNA-sequence. In a region where the two genomes do not differ, the mapped reads have the original direction and their distance coincides with the fragment length. Such a mapping is called *concordant*. If however a mapping is *discordant*, i.e., either the orientation is inconsistent or the distance differs significantly from the expected fragment size, this indicates a structural variation in the donor w.r.t. the reference.

In this paper, we only focus on deletions, i.e., a segment present in the reference is not present in - we say *deleted *from - the donor genome. Hence, paired ends spanning the deletion breakpoint in the donor genome will be mapped to the reference with a distance increased by the size of the deletion but with proper orientation. We call such a mapping *stretched*.

**Figure 1:**
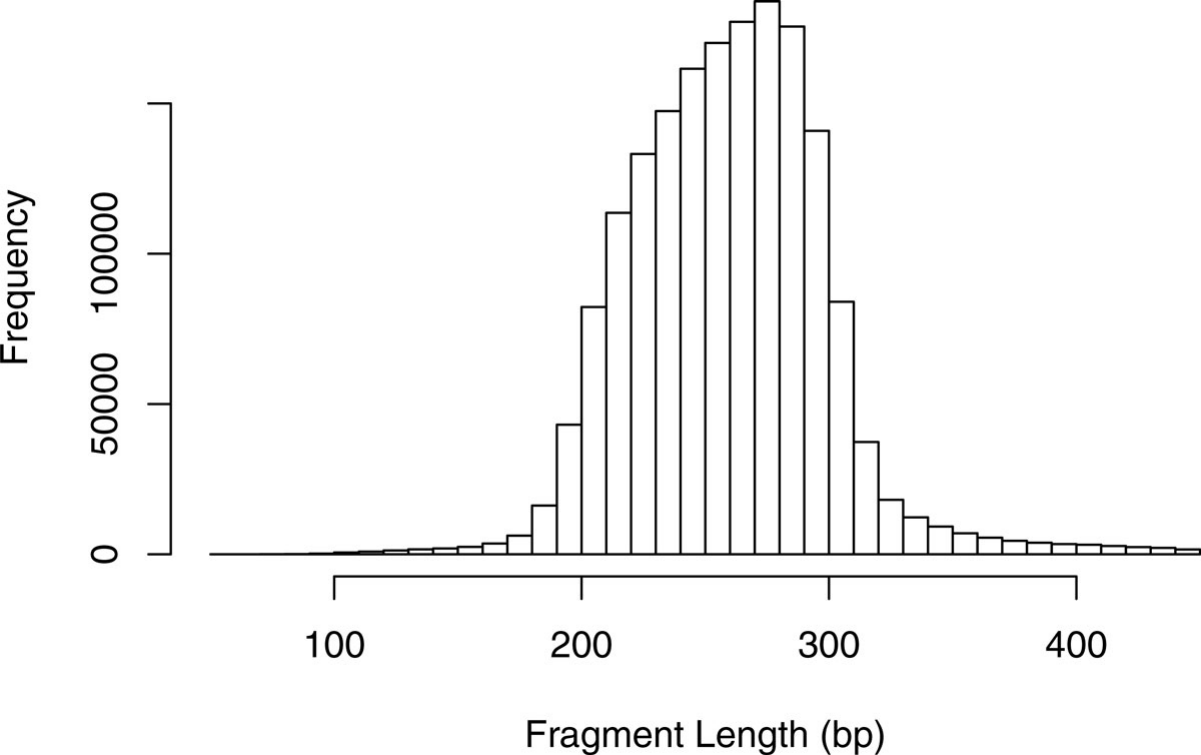
**Length distribution of the paired-end sequenced fragments**. Distribution from chromosome 21 of the sample "before treatment", discussed in Section "Real data". The length values have been extracted from the mapped paired-ends, where only unambiguously and exactly aligned pairs have been considered. Strictly speaking, also discordant mappings (those, spanning insertions or deletions) are contained. But due to their low abundance, they do not affect the distribution Significantly.

Since the fragment length is only known approximately, the size of a deletion cannot be determined exactly by paired-end mapping. It cannot even be exactly decided whether a mapping originates just from a very long fragment or is really discordant due to a deletion. One approach is to fix a minimum and maximum expected mapping distance (*D_min _*and *D_max_*), e.g., mean distance plus/minus three times the standard deviation. Then, a mapping with distance *d *is considered being stretched if *d *>*D_max_*, and the size of the putative deletion is expected to be between *d *- *D_max _*and *d *- *D_min_*. Instead of this discretization, other methods [36,37], such as ours, keep and make use of the information that the fragment length and thus the mapping distances can usually be approximated by a normal distribution with some mean *μ*. (Figure 1 shows a histogram of the fragment length distribution in the data set discussed in Section "Real data".) The expected deletion size is then also modeled to be normally distributed around *d *- *μ*.

**Figure 2:**
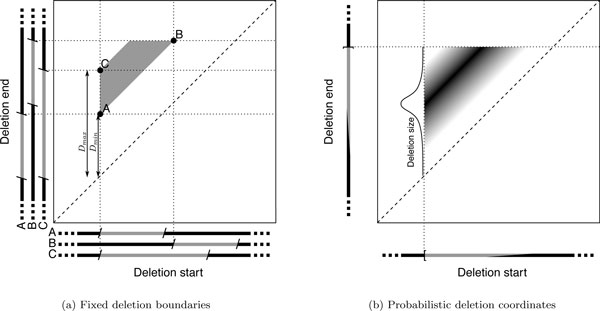
**Deletion detection by paired-end mapping**. Paired ends obtained from the donor genome (top) are mapped to the reference genome (bottom). Since the paired ends span a deletion breakpoint in the donor, the mappings are stretched. The deleted part in the reference genome is shown in gray.

Based on one of the above or a similar model, many tools, e.g. [5,6,22,33,37,38], assign all given mappings to clusters, where each cluster corresponds to one deletion that is supported by all mappings in the cluster. Figure 2 shows an example for such a deletion cluster.

## Method

In a region that possibly harbors several deletions, we obtain paired ends from the different alleles which are then all mapped to the same reference sequence. Depending on how similar the deletions are in terms of location and size, it is difficult to separate the mappings and to recover the different deletions. Our goal is to partition the set of mappings into clusters of *similar *mappings probably belonging to the same deletion. To this end, we utilize the technique of *agglomerative clustering*, which in general works as follows. At the beginning, each object (in our case stretched mapping) is a singleton cluster, and a similarity score is computed for all pairs of clusters. Then, iteratively, a pair of clusters of maximum similarity is merged, the two original clusters are replaced by this new cluster, and the similarity between the new cluster and all others is recomputed. These merging steps are repeated until either only one cluster is left, or the maximum similarity is below a certain threshold.

In the following, we will first explain how we model deletion clusters, and then introduce our similarity score, which is crucial for accurate clustering. Finally, we give some details about our implementation.

### Deletion clusters

**Figure 3:**
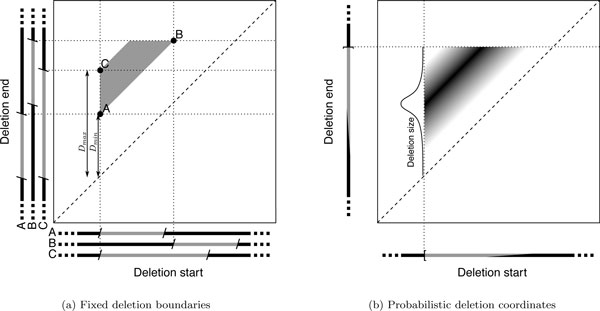
**Geometric interpretation of stretched mappings**. In both figures, the deletion start is represented on the x-axis, and the deletion end on the y-axis. (a) The coordinates for three possible deletions A, B, and C are shown exemplarily. Assuming that deletion A corresponds to the left-most and smallest possible deletion, B to the right-most, and C to the left-most and largest possible deletion, these span a trapezoid (shown in gray) which defines the coordinates of all allowed deletions, as introduced by Sindi et al. [22]. (b) Instead of assuming a smallest and largest deletion size, a probability for the deletion size and thus for the deletion coordinates is considered, represented by the shaded gray.

The similarity score used in our clustering approach follows an idea introduced by Dew et al. [23] for the analysis of mate pairs in assemblies and used for structural variation detection by Sindi et al. [22]. Based on the discretized approach explained in "Background" (minimum and maximum concordant mapping distance *D_min _*and *D_max_*), mappings and the implied location and size of the deletion are represented as a trapezoid in the two-dimensional space: The right end of the left read defines the left boundary of the start coordinate of the deletion (on the x-axis), and the left end of the right read the right boundary of the end coordinate (on y-axis). Between these, the minimum and maximum expected deletion size *d *- *D_max _*and *d *- *D_min _*span an area of "allowed" deletion coordinates. See Figure 3(a) for an example. If the trapezoids of several mappings overlap, the intersecting area (again a trapezoid) contains exactly the coordinates of all deletions supported by the mappings. The tool GASV [22] efficiently computes all maximal sets of pairwise intersecting trapezoids. Either all these clusters are output (option "maximal") or overlapping clusters are combined to single deletion calls (default). Inversions are detected by GASV in a similar fashion.

For our similarity score, we build on this two-dimensional representation of deletion coordinates, as it nicely incorporates both the location and the size of a deletion. Again, we use the x- and y-axis for the deletion start and end coordinates, respectively. But to model the deletion size, instead of the sharply bounded interval, we use a continuous distribution. As mentioned before, assuming that the fragment length follows a normal distribution with mean *μ *and standard deviation *σ*, the expected deletion size implied by a mapping with distance *d *is normally distributed around *d *- *μ *with the same standard deviation *σ*. In the geometric interpretation, for each possible start coordinate for a deletion on the x-axis, this distribution describes the expected end coordinate on the y axis. Since the deletion has to be located in-between the mappings of the paired ends, these, together with the main diagonal, define a triangle of "allowed" deletion coordinates, which is covered in the third dimension by the normal distribution as shown in Figure 3(b). In this diagram and all following, the probability will be represented by shaded gray toning.

In the clustering process, we characterize each mapping and also each cluster composed of several mappings by the above mentioned parameters: The left and right boundaries *l *and *r *for the deletion coordinates, and *μ *and *σ *of the normal distribution. We further store the number *n *of mappings in a cluster. For a singleton cluster, the parameters are directly defined by the mapping's characteristics, as described above. When we merge two mappings or two clusters *C*_1 _and *C*_2 _with parameters *l_i_, r_i_, μ_i_, σ_i _*and *n_i_*, where *i *= 1 or 2, respectively, we combine the parameters to obtain a new cluster C_1∪2 _as follows.

$$\begin{array}{ccc} n_{1\cup 2} & := & n_{1}+n_{2}\\ l_{1\cup 2} & := & \max\left\{l_{1},l_{2}\right\}\\ r_{1\cup 2} & := & \min\left\{r_{1},r_{2}\right\}\\ \mu_{1\cup 2} & := & \frac{n_{1}\mu_{1}+n_{2}\mu_{2}}{n_{1\cup 2}}\\ \sigma_{1\cup 2}^{2} & := & \frac{n_{1}\sigma_{1}^{2}+n_{2}\sigma_{2}^{2}}{n_{1\cup 2}}+\frac{n_{1}n_{2}{\left(\mu_{1}-\mu_{2}\right)}^{2}}{n_{1\cup 2}^{2}} \end{array}$$

With our choice of *l *and *r *as the maximum and minimum, respectively, we follow an "average link" strategy as it corresponds to taking the intersection of the triangles. This is the most intuitive way since the new cluster should represent the "area" of deletion coordinates to those which are compatible to both of the original clusters. The equation for the joint standard deviation is borrowed from population-based statistics for aggregating non-overlapping sub-populations. If the two clusters agree in the location of the deletion, i.e., *μ*_1 _- *μ*_2 _= 0, the resulting $\sigma_{1\cup 2}^{2}$ is the mean of the two standard deviations. The more the two clusters disagree, the broader becomes the distribution. For reasons we will explain later, this way of joining the deviations turned out to be better suitable for our similarity score and the resulting performance in clustering than using the actual joint variance.

### Similarity score

For the clustering, we need to define a score function that, for a given pair of clusters, defines a value - the more similar the clusters are, the higher this score. Our similarity score is defined in the range from zero to one. In our case, the similarity of clusters, i.e. deletion predictions, depends on two factors: The location and the size of the deletion, both included in our geometric cluster model. Recall that each cluster *C *defines a volume, say V(*C*), whose base is given by a triangle with a Gaussian shaped "mountain" on top. The larger the overlap of two triangles, the more they agree in the predicted location of the deletion. The closer the ridge of their mountains, the more they agree in the deletion size. Based on this observation, we define the score as the normalized intersection of the two volumes.

$$\text{sim}\left(C_{1},C_{2}\right):=\frac{\text{V}\left(C_{1}\right)\cap \text{V}\left(C_{2}\right)}{\max\left\{\text{V}\left(C_{1}\right),\text{V}\left(C_{2}\right)\right\}}$$

This score has the following properties.

• The score is zero if and only if the triangles do not overlap, which means that there is no location for a deletion compatible with both clusters.

• The score is one if and only if the two clusters are equal, because, only if all parameters of the two clusters are equal, the intersection volume equals the maximum.

• It is more sensitive to differences in the deletion size than in the location. The reason for this is that a shift parallel to the ridge of the volume does not affect the intersection volume to such an extent as a shift perpendicular to it. This is a particularly desired behavior since we expect a deletion breakpoint being covered by several mappings. In some mappings the breakpoint lies more to the left and in others more to the right, which - even if all mappings are correct - corresponds to staggered triangles. Such triangles, even if staggered, would have to be clustered and should thus not be punished too hard by a low score. In contrast, a difference in the deletion size indicates a disagreement of the mappings.

We now come back to the issue how to combine the standard deviations of several mappings. As already mentioned, we did not choose the more intuitive combination by computing the standard deviation of the means, i.e. $\sigma_{1\cup 2}^{2}:=\sigma^{2}/n_{1\cup 2}$, where *σ *is the standard deviation of the fragment length. This results in narrower distributions for clusters containing more mappings. On the one hand, this would make sense since the prediction is more accurate, the more mappings support it. On the other hand, besides computational problems due to score values close to zero, this also yields an artifact: Clusters containing only a few mappings have a broader volume and are thus more likely to be clustered than larger clusters with a narrow volume. The order in which clusters are aggregated would be dominated by their cardinality, rather than by their similarity. To avoid this, we chose our definition of $\sigma_{1\cup 2}^{2}$ to be independent of the cardinality of the clusters.

It remains to describe the stopping criterion for the clustering process. Since the normal distribution is never exactly zero, any two clusters that overlap in their base (triangle) have a non-empty intersection volume, no matter how weak the overlap is. We thus set a minimum threshold *S_min _*for the similarity score. If no pair of clusters has similarity larger than *S_min_*, the clustering process is stopped. To avoid the introduction of a parameter which is arbitrarily fixed or has to be given by the user, we examine the data in a preprocessing step to determine a threshold that separates Significant similarity values from noise. For each mapping, we determine the smallest non-zero similarity score to any other (overlapping) mapping. Setting *S_min _*to the median of these minima showed robust accurate behavior in practice, also approved in our experiment as explained at the end of "Simulation of overlapping deletions".

### Implementation

In general, the run time complexity of agglomerative clustering depends on the similarity score. Using priority queues to store the scores, *O*(*N*^2 ^log *N*) pairwise score computations have to be performed to cluster *N *objects. Our score can be computed as follows. In each triangle, the height of the volume is constant on a line parallel to the main diagonal and thus parallel to the hypotenuse of the triangle. This allows us to compute the volume by traversing a triangle (or the intersection of two triangles, which is again a triangle) starting from its hypotenuse towards the upper left corner, and summing up the product of the length of the line and the height of the volume given by the normal distribution (or the minimum of two, respectively). Since this takes *O *(*r *- l) time and the score computation consists of a constant number of volume computations, the overall run time complexity of the agglomerative clustering is in *O *(*L N*^2 ^log *N*), where *L *is the maximum length of a mapping.

In practice, the run time is dominated by reading the input and thus approximately linear in the number of mappings.

We implemented the method in JAVA. The input consists of one or several BAM files and a simple tabular separated file listing the mean segment length and standard deviation for each file. Additionally, a color can be specified for each BAM file, which is used to visualize the mappings in the graphical output. The mappings are read from the BAM files using SAMtools [39] and applying several filters. Only mappings of high quality (quality value at least 20), without gaps, uncapped and without co-optimal mapping locations are used. Further, the mappings can be filtered by their length. Here, a minimum length of mean plus three times standard deviation showed good performance in practice.

Before the actual clustering, we partition the mappings into so-called *regions*, maximal subsets such that no mapping from one set overlaps with any mapping in another subset. These regions are then clustered independently.

**Figure 4:**
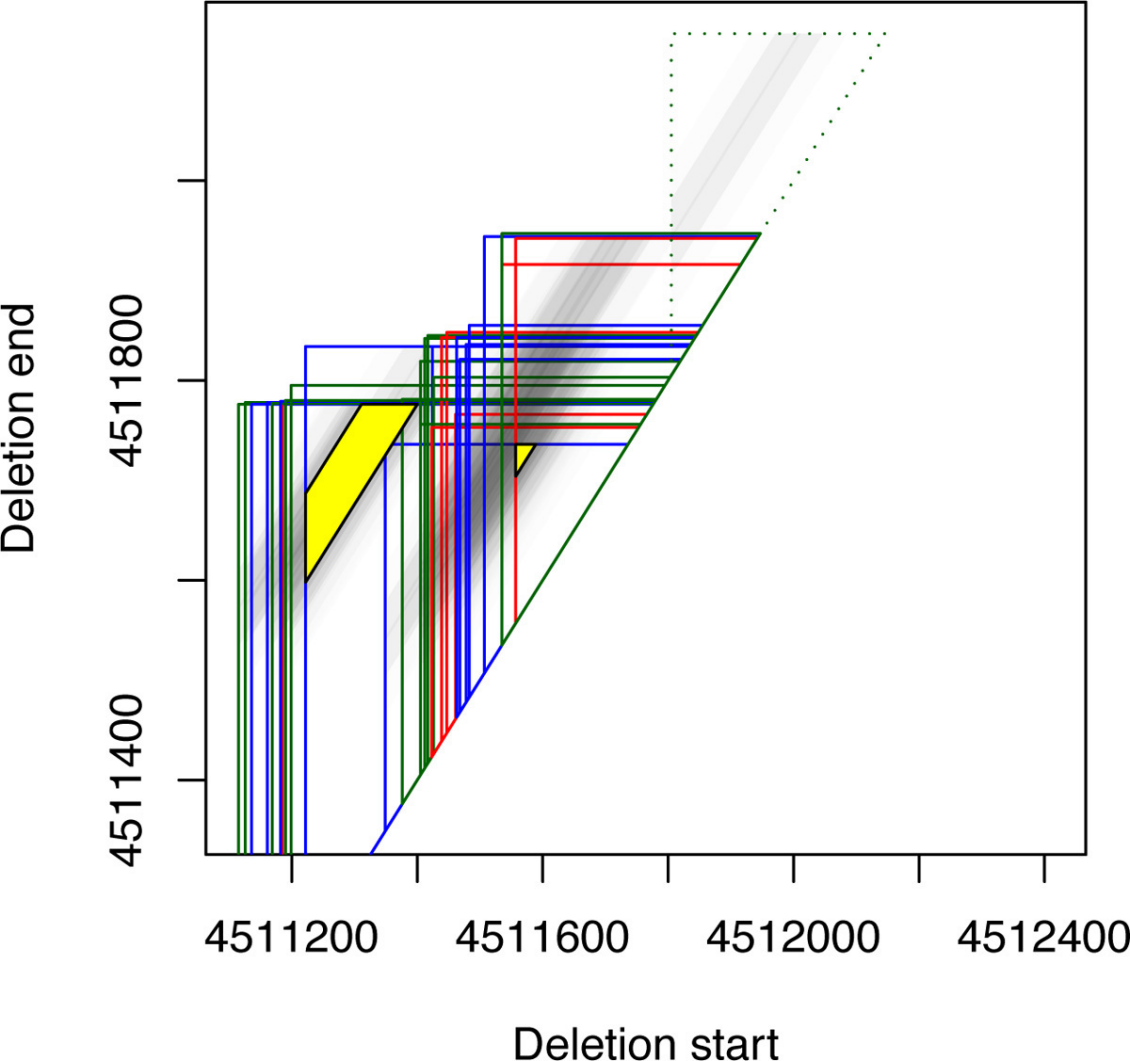
**Region on chromosome 19 possibly harboring two overlapping deletions**. The mappings of the different data sets (before treatment, after treatment, and relapse) are represented as red, blue and green triangles, respectively, where the third dimension is indicated by shaded gray tones. The detected deletions are shown as yellow trapezoids. A singleton cluster, which is probably an erroneous mapping, is depicted with dotted lines.

The output is composed of two parts. (1) A tabular separated file is generated, listing all detected clusters including, besides other details, the breakpoint regions, the mean expected deletion size and the standard deviation, and number of mappings from each BAM file. The latter information can for instance be used in a post-processing step to determine whether a deletion is patient specific: If several samples have been analyzed, for instance "healthy" and "tumor", a cluster with a high number of mappings from the tumor set but a small number of mappings from the healthy set indicates a tumor-specific deletion. Additional file 1 lists the mappings for each cluster. (2) A graphical output of each region is provided in form of R-code, which produces PDF graphics as can be seen in Figure 4. The triangle for each mapping is shown in the color assigned to the corresponding BAM file, and the third dimension is indicated by shaded gray tones. Clusters with a sufficient number of mappings (threshold chosen by the user, default 2) are highlighted by a yellow trapezoid (spanned by *l, r, μ*-3*σ*, and *μ*+3*σ*) labeled with the number of mappings. Smaller clusters are not shown and their mappings are depicted by dotted lines. The R-code can easily be modified to produce customized scalable vector graphics.

The tool, including the source code and example data, is available from the Bielefeld University Bioinformatics Server: http://bibiserv.cebitec.uni-bielefeld.de/agglodel/

## Results and discussion

Before we present results on real data from three samples of a cancer patient, we first investigate the accuracy of the new clustering method in distinguishing overlapping from single deletions in simulated data sets.

### Simulation of overlapping deletions

Since, to the best of our knowledge, no other method exists that aims at identifying overlapping deletions, and because such instances are difficult to detect or verify in the wet lab, there is no gold standard available we could use for an evaluation. Instead, we created a simulated data set which is based on previously detected single deletions.

**Figure 5:**
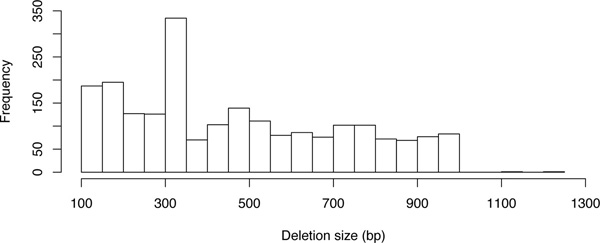
**Distribution of the deletion size used for sampling deletions in the simulation-based evaluation**.

We took chromosome one of the human genome (hg19, GRCh37) and created two diploid copies of it into which we introduced deletions. From a list of variations downloaded from the *Database of Genomic Variants *[[40], chr1 of indel.hg19.v10.nov.2010.txt], we first sampled 1,000 non-overlapping deletions. For 500 of them, we simulated an overlapping deletion, the size of which was sampled from the afore mentioned list. Paired-end mapping approaches are not suited to detect small deletions. Thus, on the one hand, we only sampled deletions of a certain minimum length. On the other hand, we could not set this threshold too high to be able to sample enough non-overlapping deletions in the first run. A minimum length of 105 turned out to be a good tradeoff. The length distribution is shown in Figure 5.

**Figure 6:**
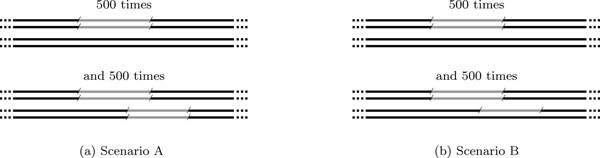
**Two scenarios used for sampling deletions in the simulation-based evaluation**. Two diploid chromosomes with 500 non-overlapping deletions and 500 pairs of overlapping deletions (both homozygous, and homozygous and heterozygous) have been simulated.

From these artificial chromosome copies, we sampled Illumina paired ends using the tool SimSeq [41]. We selected a typical read length of 100, and to be comparable to the data set we analyze in Section "Real data", we chose a mean fragment lengths of 300 with standard deviation 35. We performed several runs with different coverage for the different alleles to simulate the two scenarios shown in Figure 6: (A) The overlapping deletions are both homozygous, and (B) one deletion is homozygous and the other is heterozygous. Both settings were simulated using 20× and 60× sequence coverage, the first of which roughly corresponds to the real data set (Section "Real data"). We mapped the obtained reads back to the original hg19 sequence with the mapping software BWA [42].

We used our agglomerative clustering approach with the filtering of mappings as explained in "Method", and also ran two other tools on the same set of filtered mappings. First of all, we included GASV [22] (release 2.01) into our evaluation, as our similarity score is based on an extension of the geometric interpretation used there. We set the minimum cluster cardinality to 2 and used both, the option to output all maximal sets of overlapping trapezoids (see "Method") which we will refer to as GASV_max, and the default to output merged clusters, referred to as GASV. Although a follow-up version GASVPro [33] has been published recently, the available software does not yet include all necessary preprocessing tools to process BAM files. Secondly, we also ran CLEVER [37] as one of the most recent and accurate variation detection tools. This method computes a probability for mappings arising from the same deletion, and builds a graph with the mappings as vertices and edges for pairs with Significantly high probability. Clusters are determined as maximal cliques in this graph, which in general allows overlapping deletions in the output. Clusters are determined as maximal cliques in this graph.

**Figure 7:**
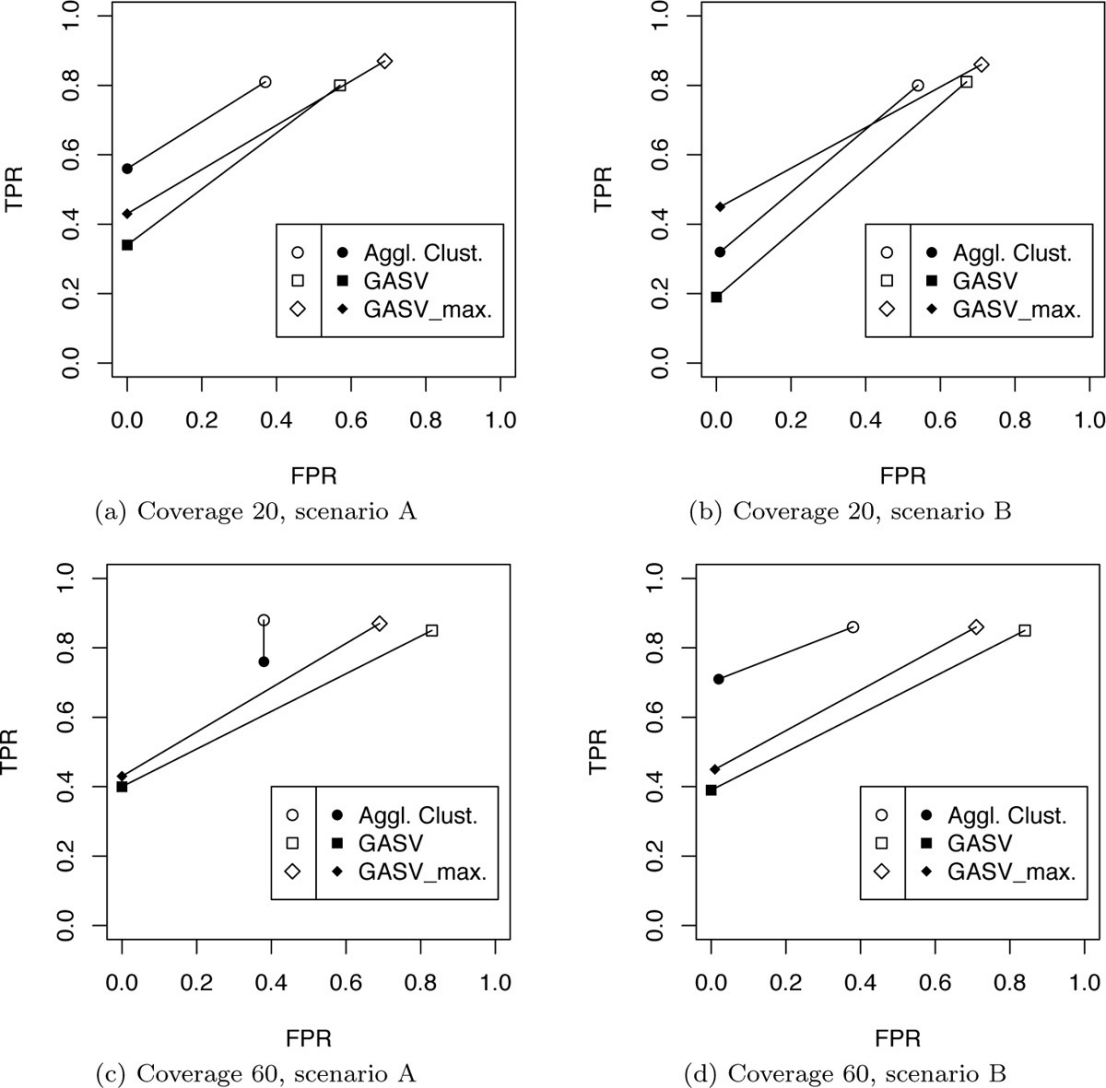
**Accuracy of different methods in detecting single or overlapping deletions**. For different coverages (20× and 60×), and two different simulated scenarios (see Figure 6), the accuracy in distinguishing single from overlapping deletions has been measured. The empty symbols represent the true and false positive rate for detecting single deletions (TPR_1_, FPR_1_). The solid symbols represent the true and false positive rate for detecting pairs of overlapping deletions (TPR_2_, FPR_2_).

The results are detailed in the table in the appendix. In Figure 7, we summarize the results in form of ROC plots showing the accuracy at identifying non-overlapping deletions and pairs of overlapping deletions in terms of true and false positive rates:

$$\begin{array}{cc} \text{TP}{\text{R}}_{1}=\frac{\text{T}{\text{P}}_{1}}{{\text{P}}_{1}}=\frac{\#\text{correctly predicted single del's}}{\#\text{simulated single del's}} & \text{FP}{\text{R}}_{1}=\frac{\text{F}{\text{P}}_{1}}{{\text{N}}_{1}}=\frac{\#\text{falsely predicted single del's}}{\#\text{sim}\text{. pairs + }\#\text{no del}\text{. detected as del}\text{.}}\\ \text{TP}{\text{R}}_{2}=\frac{\text{T}{\text{P}}_{2}}{{\text{P}}_{2}}=\frac{\#\text{correctly predicted pairs}}{\#\text{simulated pairs}} & \text{FP}{\text{R}}_{1}=\frac{\text{F}{\text{P}}_{2}}{{\text{N}}_{2}}=\frac{\#\text{falsely predicted pairs}}{\#\text{sim}\text{. single del's + }\#\text{no del}\text{. det}\text{. as del}\text{.}} \end{array}$$

On average, CLEVER detected 92% of all deletions and made only about 1% wrong predictions. Even though maximal cliques output by CLEVER can be overlapping and the output can thus contain overlapping deletion predictions, this has to be understood as a technical consequence of the clustering procedure as CLEVER does not aim at predicting overlapping clusters. Nevertheless, we tried to distinguish between non-overlapping and overlapping deletions by simply considering non-overlapping cliques and pairs of overlapping cliques. The true and false positive rates were below 0.3 for all scenarios (not shown in the figure).

In all settings, agglomerative clustering with the proposed similarity score proves to be accurate. GASV performs well, where using the option to output all clusters before merging turned out to be slightly more effective in this setting.

**Figure 8:**
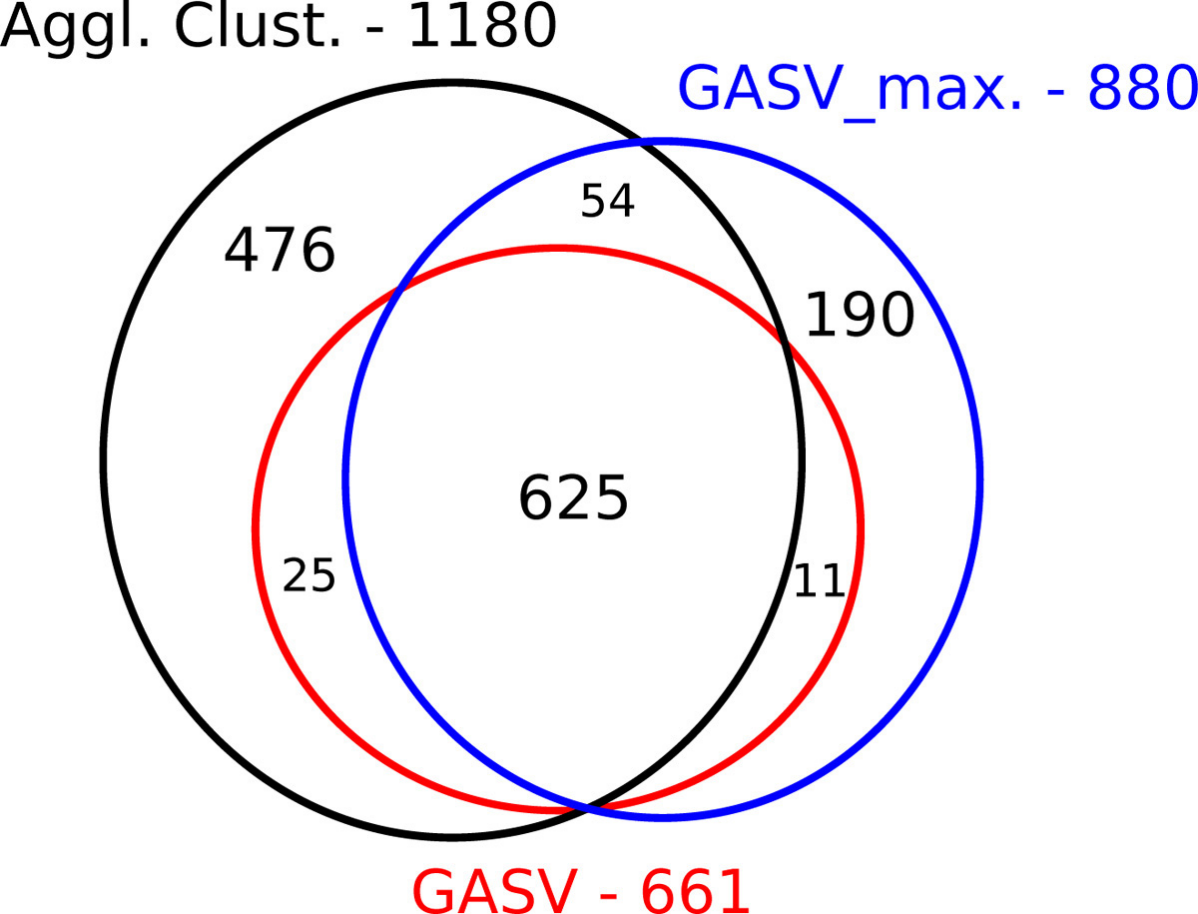
**Identification of overlapping deletions**. This Venn diagram shows how many pairs of overlapping deletions have been correctly identified by the different tools an all four simulation settings. Note that the areas are not scaled exactly proportional.

**Figure 9:**
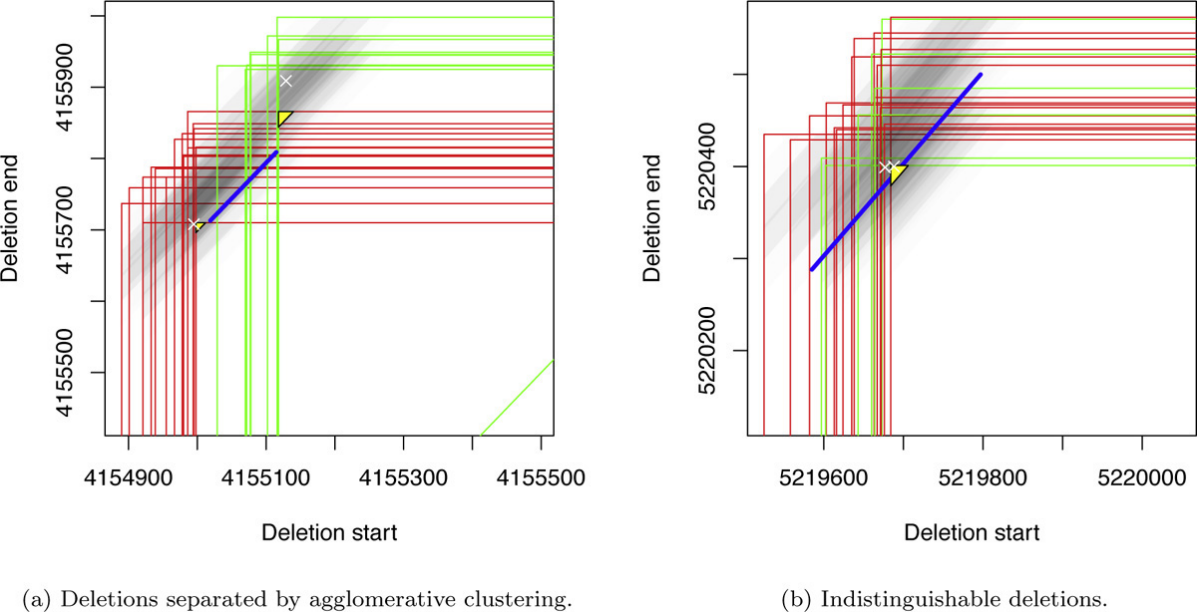
**Two instances of the evaluation**. The green and red triangles (shown partially) represent the mappings of the two alleles, and the third dimension is represented in shaded gray tones. The true deletion coordinates are indicated by white crosses, deletions obtained by agglomerative clustering are depicted in yellow, and the blue line shows the breakpoint region predicted by GASV.

As can be seen in the Venn diagram in Figure 8, overall, 625 pairs of overlapping deletions have been correctly identified by all three approaches, 476 were exclusively found by our approach, none by GASV, and 190 by GASV_max. Figure 9(a) shows a typical example in which agglomerative clustering correctly unraveled two overlapping deletions, whereas GASV (both variants) makes one prediction that is rather vague. Figure 9(b) exemplifies that in some instances, the two overlapping deletions were so similar in location and size that they appear to be indistinguishable. The mean distance (all scenarios) of simulated overlapping deletions falsely detected as one deletion was about 20% smaller than those correctly detected as two deletions; the ratio for the deletion sizes was about the same. Further, the resolution was worse for smaller deletions: Falsely classified deletions were on average 13% smaller than correctly classified ones.

We also investigated, when two overlapping deletions have been called correctly, how accurate the mappings originating from the two different alleles have been assigned to the two clusters. Ideally, one clusters should only contain mappings from the first deletion and the other cluster only those from the second deletion. On those pairs, correctly identified by all three methods, GASV clusters contained 0.041% mis-assigned mappings, GASV_max 0.019%, and our clusters contained 0.006%. In total, i.e., including the more difficult cases GASV did not detect as pairs at all, we observed 1.3% mis-assignments in our clusters and 4.0% in the GASV_max clusters (averaged over all four simulation settings).

**Figure 10:**
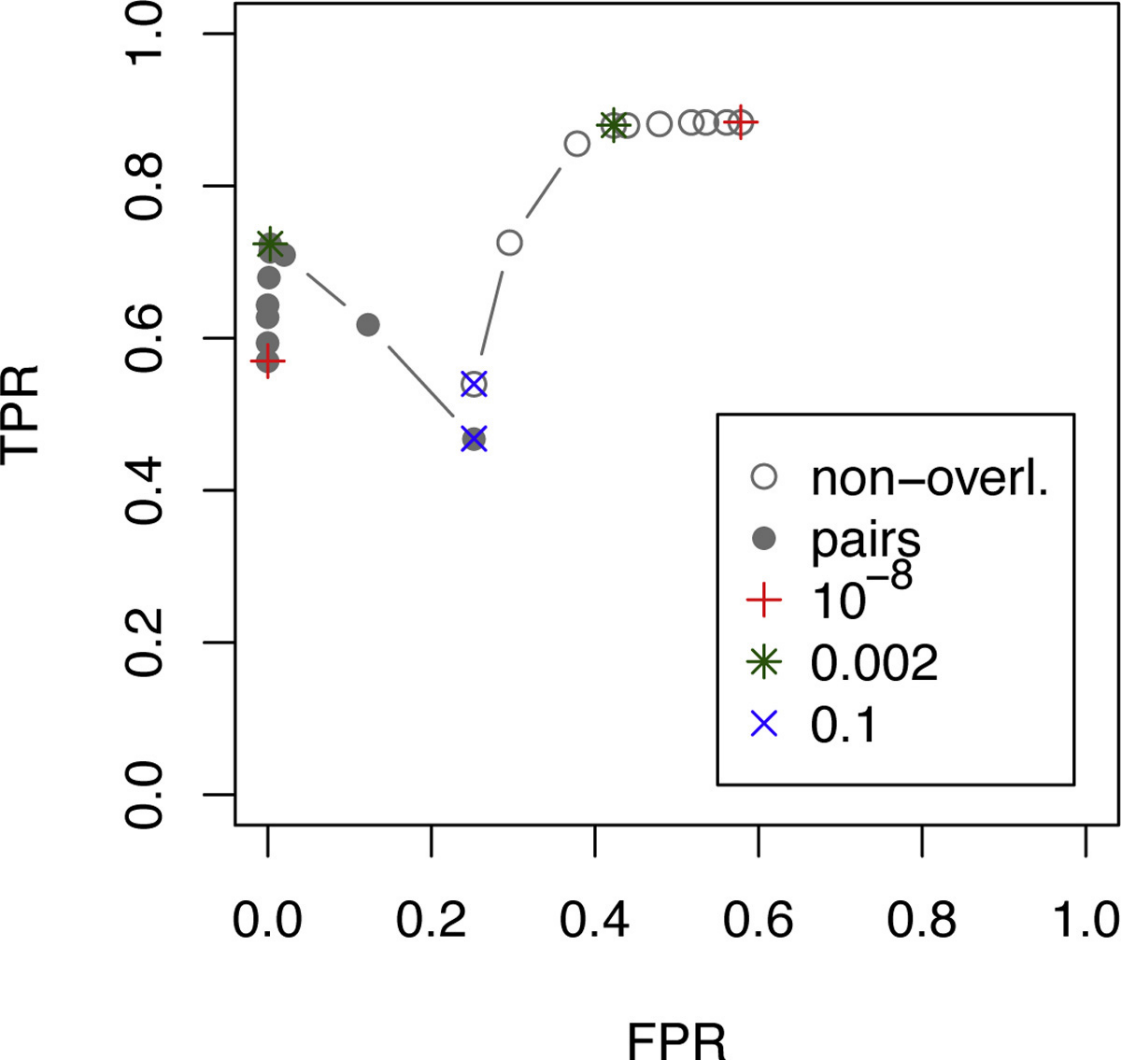
**Accuracy depending on the similarity score used as threshold in the agglomerative clustering**. Thresholds 10^-8^, 10^-7^, . . ., 10^-3^, 0.002, 0.01, 0.05, and 0.1 have been tested for scenario B (see Figure 6(b)) and coverage 60×. The default threshold as computed from the data was approximately 0.002, marked with a *. The minimum and maximum thresholds are marked with a + and ×, respectively. The true and false positive rates for non-overlapping and pairs of overlapping deletions are shown.

To analyze the similarity threshold used as a stopping criterion in our clustering, we performed several runs with varying thresholds and summarized the performance in a ROC curve, shown in Figure 10. In this simulation setting, the threshold that is computed from the data as described in Section "Method" turns out to be close to optimal in terms of accurately detecting non-overlapping deletions and even optimal for pairs of overlapping deletions.

### Real data

The Department of Paediatric Oncology, Haematology and Immunology at the Düsseldorf University Hospital, Germany, provided sequencing data of an acute lymphoblastic leukemia patient. Three samples (before treatment, after treatment, and relapse) have been sequenced on an *Illumina HiSeq 2000 *with read length 51, segment length around 300, and sequence coverage of 6×, 8× and 8×, respectively. At the Institute of Medical Informatics at University of Münster, Germany, the reads have been mapped to hg19 with BWA [42] (version 0.5.9, at most 3 mismatches and standard parameters otherwise) and Picard [43] has been used to remove duplicates. Figure 1 exemplarily shows the fragment length distribution for chromosome 21 from one of the data sets estimated from the mapping distances. It approves that the assumption of a normal distribution is approximately met.

Processing the data on a *Sun Fire X4600 M2 *with dual-core AMD processors at 2.6 GHz and 32 GB RAM took in total about 8:46 CPU hours, from which 7:56 hours were needed to read and filter the 67 GB of input, and only 50 minutes were necessary to cluster the filtered 3,249,648 mappings, and generating the output. We considered all mappings with distance at least three standard deviations larger than the mean. Those have been filtered and partitioned into 731,522 regions as described in "Method", only 288,552 of which contained at least two mappings. In total, 101,910 deletions supported by at least two mappings have been detected, comprising 104 pairs of overlapping deletions. Table 1 gives an overview of the number of deletions, and the amount of overlaps found.

**Table 1:** Number of overlapping deletions.

Size (# deletions)	0	1	2	3	4	5	6	7	8	9	≥ 10	Total
# regions	631,769	97,869	1,768	68	25	8	2	2	3	4	4	731,522
# components	--	101,615	104	9	4	3	1	2	0	1	0	101,739

If the breakpoint region of a deletion, i.e., the interval defined by *l *and *r *of the cluster, overlaps another breakpoint region, these deletions are assigned to the same *component*. That means, non-overlapping deletions build components of size one, pairs of overlapping deletions build components of size two, etc. Note that deletions found in one *region *(cf. Section "Method") do not necessarily build one component. Below, the number of regions and components for different sizes are given.

Many highly overlapping deletions were found in telomeric or centromeric regions, where mappings are not very reliable. Figure 4 exemplarily shows a region where we found overlapping clusters. The shown region is also an example for a general observation we made: Agglomerative clustering with our similarity score nicely identifies outliers among the discordant mappings, as those remain as singleton clusters. In our approach, such putatively erroneous mappings do not distort the main deletion prediction, whereas, for instance in GASV, a single mapping can drastically affect a cluster if the corresponding trapezoid overlaps it, or can result in many maximal sets of overlapping trapezoids.

An experimental validation of this and further findings is currently being performed. Here, we wanted to give an overview of the general performance of the presented method on a real world instance. In cooperation with the Düsseldorf University Hospital, further analyses of this and other data sets are planned.

## Conclusions

It is well known that structural variations in the human genome play an important role in the development of diseases, especially cancer. In particular, the accurate detection of deletions still remains a challenging task. A preliminary study has motivated that detection methods should be capable of handling overlapping deletions to be able to draw a clearer picture of variations in heterogeneous samples, e.g., to distinguish cancer from patient specific mutations.

In the present study, we have demonstrated that agglomerative clustering is suitable for this task. We have introduced a similarity score that is based on a geometric and probabilistic interpretation of paired ends which have been mapped to a reference sequence. Taking into account both the location and the length of a deletion, this scoring allows to effectively cluster mappings into possibly overlapping clusters. The method has successfully been applied on real data, and has proven to be accurate according to a simulation-based evaluation.

Here, we have investigated the performance of an intuitive clustering approach. On the one hand, the simplicity of agglomerative clustering offered us insight into the clustering process and understanding of its behavior. On the other hand, computationally more sophisticated clustering strategies could perform even better, if they are computationally not too expensive for such large data sets.

We believe that there is still more potential to be explored in the detection of structural variants, especially deletions. Besides the combination with other approaches, such as split read or coverage analysis, the increasing availability of several samples from one patient offers a dimension that has to be investigated.

### Note added in proof

Another clustering based method to call deletions (and insertions) from paired-end mapping data from heterogeneous samples has been published very recently. The tool SVM^2 ^by Chiara et al. [44] utilizes a support vector machine incorporating, similar to our tool, mapping location and mapping distance, but also coverage information. Including SVM^2 ^into our simulation-based evaluation could not be finished during revision of the present manuscript.

## Competing interests

The author declares that they have no competing interests.

## Authors' contributions

R.W. developed, implemented and evaluated the method, and wrote the paper.

## Declarations

The publication costs for this article were funded by the Deutsche Forschungsgemeinschaft and the Open Access Publication Funds of Bielefeld University Library.

This article has been published as part of *BMC Genomics *Volume 14 Supplement 1, 2013: Selected articles from the Eleventh Asia Pacific Bioinformatics Conference (APBC 2013): Genomics. The full contents of the supplement are available online at http://www.biomedcentral.com/bmcgenomics/supplements/14/S1.

## Supplementary Material

Additional file 1 **Results of the simulation-based evaluation**. Results of the simulation-based evaluation described in Section "Simulation of overlapping deletions" of the paper. The leftmost column specifies the number of simulated overlapping deletions (no deletion, single deletion, or pair of overlapping deletions) and the predictions (no deletion, single deletion, pair of overlapping deletions, or three or more overlapping deletions). The remainder of the table shows the corresponding counts per tool (agglomerative clustering, GASV [22], GASV with option "maximal", and CLEVER [37]) for four different settings (coverage 20× and 60×, and scenario A and B, cf. Figure 6). Click here for file
