# Correction: Unraveling overlapping deletions by agglomerative clustering

**DOI:** 10.1186/1471-2164-14-S1-S16

**Published:** 2013-03-15

**Authors:** Roland Wittler

**Affiliations:** 1 Genome Informatics, Faculty of Technology and Institute for Bioinformatics, Center for Biotechnology (CeBiTec), Bielefeld University, 33594 Bielefeld, Germany

## 

After the publication of my article [1], I noticed that Figure 2 shows the wrong graphic and Figure 7 was incorrect. The correct figures can be found here.

**Figure 2:**
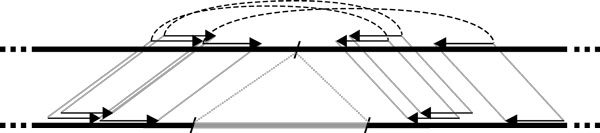
**Deletion detection by paired-end mapping**. Paired ends obtained from the donor genome (top) are mapped to the reference genome (bottom). Since the paired ends span a deletion breakpoint in the donor, the mappings are stretched. The deleted part in the reference genome is shown in gray.

**Figure 7:**
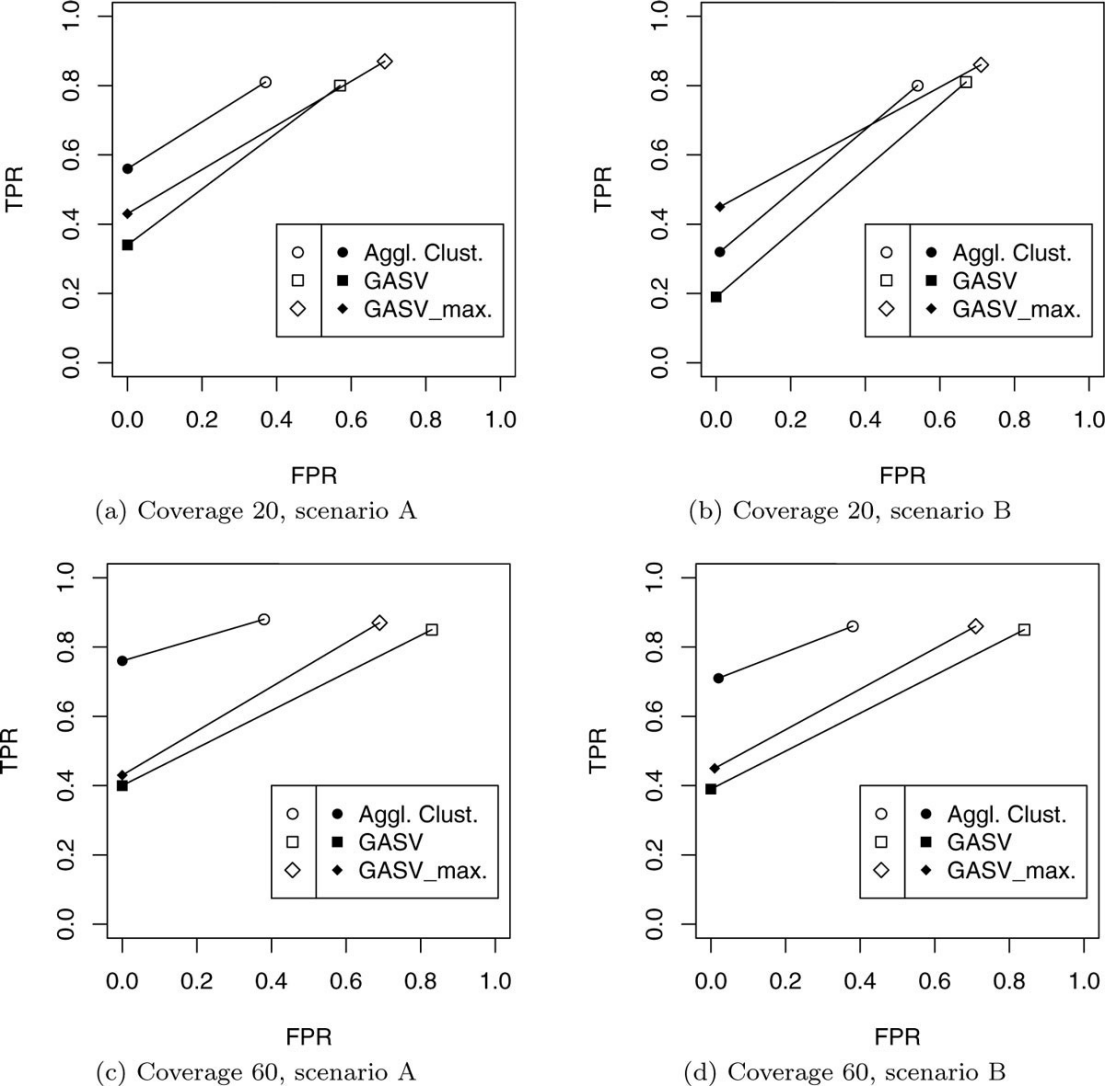
**Accuracy of different methods in detecting single or overlapping deletions**. Accuracy of different methods in detecting single or overlapping deletions. For different coverages (20× and 60×), and two different simulated scenarios (see [[1], Figure 6]), the accuracy in distinguishing single from overlapping deletions has been measured. The empty symbols represent the true and false positive rate for detecting single deletions (TPR1, FPR1). The solid symbols represent the true and false positive rate for detecting pairs of overlapping deletions (TPR2, FPR2).
