# Supplementary material for: Unraveling overlapping deletions by agglomerative clustering
Source: BMC Genomics. 2013 Jan 21;14(Suppl 1):S12. doi: 10.1186/1471-2164-14-S1-S12 (PMC3549816; doi:10.1186/1471-2164-14-S1-S12)
Supplement: Additional file 1 — Results of the simulation-based evaluation. Results of the simulation-based evaluation described in Section "Simulation of overlapping deletions" of the paper. The leftmost column specifies the number of simulated overlapping deletions (no deletion, single deletion, or pair of overlapping deletions) and the predictions (no deletion, single deletion, pair of overlapping deletions, or three or more overlapping deletions). The remainder of the table shows the corresponding counts per tool (agglomerative clustering, GASV [22], GASV with option "maximal", and CLEVER [37]) for four different settings (coverage 20× and 60×, and scenario A and B, cf. Figure 6). [file 1471-2164-14-S1-S12-S1.pdf]

Additional file 1 — Results of the simulation-based evaluation

Results of the simulation-based evaluation described in Section "Simulation of overlapping deletions" of the paper. The leftmost column specifies the number of simulated overlapping deletions (no deletion, single deletion, or pair of overlapping deletions) and the predictions (no deletion, single deletion, pair of overlapping deletions, or three or more overlapping deletions). The remainder of the table shows the corresponding counts per tool (agglomerative clustering, GASV [1], GASV with option "maximal", and CLEVER [2]) for four different settings (coverage 20× and 60×, and scenario A and B, cf. Figure 6).

	Coverage 20×, Scenario A				*Coverage 20×, Scenario B*			
sim.→pred.	Aggl.	GASV	GASV_max	CLEVER	Aggl.	GASV	GASV_max	CLEVER
0→1	13	113	782	113	9	115	796	93
0→2	0	0	3	3	0	0	6	1
0→≥3	0	0	7	0	0	0	4	0
1→0	95	98	58	60	96	97	57	60
1→1	405	402	434	144	399	403	430	145
1→2	0	0	2	180	5	0	5	181
1→ ≥3	0	0	6	116	0	0	8	114
2→0	43	93	27	26	71	102	30	30
2→1	175	236	110	32	266	300	127	55
2→2	282	170	217	62	162	97	223	115
2→ ≥3	0	1	146	380	1	1	120	300

	Coverage 60×, Scenario A				*Coverage 60×, Scenario B*			
sim.→pred.	Aggl.	GASV	GASV_max	CLEVER	Aggl.	GASV	GASV_max	CLEVER
0→1	168	1350	782	8	155	1401	796	9
0→2	0	0	3	0	0	0	6	1
0→ ≥3	0	0	7	0	0	0	4	0
1→0	58	75	58	51	58	77	57	51
1→1	439	425	434	17	429	423	430	18
1→2	3	0	2	40	12	0	5	39
1→ ≥3	0	0	6	392	1	0	8	392
2→0	27	113	27	22	30	106	30	22
2→1	87	187	110	7	94	198	127	11
2→2	381	199	217	10	355	195	223	8
2→ ≥3	5	1	146	461	21	1	120	459

References

1. Sindi S, Helman E, Bashir A, Raphael B: **A geometric approach for classification and comparison of structural variants**. *Bioinformatics* 2009, **25**(12):i222–i230. [Proc. of RECOMB 2009].
2. Marschall T, Costa I, Canzar S, Bauer M, Klau G, Schliep A, Schönhuth A: **CLEVER: Clique-Enumerating Variant Finder**. *Bioinformatics* 2012.
